# 4447 Leptin supplementation prevents the loss of hypoglycemia-induced glucagon release following exposure to six days of severe caloric restriction in mice

**DOI:** 10.1017/cts.2020.76

**Published:** 2020-07-29

**Authors:** David H McDougal, Marina A. DuVall, Christopher D. Morrison, Laura A. Moldovan, Rajvi Jariwala

**Affiliations:** 1 Pennington Biomedical Research Center- LA CaTS

## Abstract

OBJECTIVES/GOALS: We have recently shown that mice exposed to six days of 60% caloric restriction acutely display reduced hypoglycemia-induced glucagon release following refeeding, and that this effect is concurrent with low leptin levels. The current study was conducted to ascertain if leptin treatment during caloric restriction would reverse this effect. METHODS/STUDY POPULATION: Three groups of mice were used, an ad libitum (Ad-lib) fed group and two caloric restriction (CR) groups, one of which received twice daily leptin injection (0.5-1μg/g; IP) and the other vehicle (saline) during their caloric restriction. CR mice were placed on 60% caloric restriction for 6 consecutive days. Ad lib mice were housed in an identical manner but fed ad libitum during this same period. Following 6 days of restriction, CR mice were given ad lib access to food for 16 h. After the 16 h period of refeeding, both CR and ad lib mice began a 6 h fast which was immediately followed by a hypoglycemic insulin tolerance test (ITT). ITTs consisted of a variable dose of insulin intended to achieve a blood glucose of ~45 mg/dL within 60 minutes, at which time blood was collected for glucagon and corticosterone assays. RESULTS/ANTICIPATED RESULTS: The mean blood glucose levels during the ITT at 45 and 60 minutes post injection across all three groups were 46.8 + 3.1 and 37.0 + 2.4, respectively. There were no significant differences in glucose levels between the three groups at these two time points. As expected, saline treated CR mice displayed significantly reduced serum glucagon levels in response to the ITT relative to Ad-lib mice (23.5 + 10.9 vs. 91.7 + 20.8 pg/mL, p = 0.009). In contrast, leptin-treated CR mice maintained their hypoglycemia-induced glucagon response to the ITT (78.0 + 16.8 pg/mL, p>0.99 vs. Ad-lib group). In addition, although corticosterone levels in saline treated CR mice were numerically lower than in Ad-lib mice, this difference was not statistically significance (3928 + 277 vs. 4571 + 178 pg/mL, p = 0.179). DISCUSSION/SIGNIFICANCE OF IMPACT: Diabetes patients on insulin therapy often develop impaired hypoglycemic counter-regulation which can lead to life-threatening hypoglycemic complications. Our results suggest that leptin may hold promise as a therapeutic intervention for the prevention of impaired hypoglycemic counter-regulation in persons with diabetes.

